# Health care costs and service utilization in the first year following moderate to severe traumatic injury

**DOI:** 10.1186/s12913-024-12016-6

**Published:** 2024-12-03

**Authors:** Mari Storli Rasmussen, Yuan Zhang, Nada Andelic, Eline Aas

**Affiliations:** 1 Dept. Physical Medicine & Rehabilitaion, Oslo University Hospital, Oslo, Norway; 2 Faculty of Health Sciences, Oslo Metropolitan University, Oslo, Norway; 3 Department of Health Management and Health Economics, Institute of Health and Society, Faculty of Medicine, University of Oslo, Oslo, Norway; 4 Research Centre for Habilitation and Rehabilitation Models and Services (CHARM), Institute of Health and Society, Faculty of Medicine, University of Oslo, Oslo, Norway; 5 Division for Health Services, Norwegian Institute of Public Health, Oslo, Norway

**Keywords:** Traumatic injury, Health care utilization, Health care costs

## Abstract

**Background:**

Most of the previous studies on costs following trauma have focused solely on in-hospital costs and costs associated with the acute treatment and early rehabilitation. As a result, post-hospital costs are often neglected in the estimation of total costs. We aimed to describe service utilization and total costs for health care services, rehabilitation services, and social support in the periods 0–6 months and 7–12 months after moderate-to-severe traumatic injury. Further, we explored costs and their associations with sociodemographic, clinical and injury-related variables.

**Methods:**

Data were obtained from a prospective, 12 months follow-up study of patients in all ages with moderate-to-severe traumatic injury determined by a New Injury Severity Score (NISS) > 9, admitted directly or within 72 h to the trauma referral centres in year 2020. Data on utilization of health care and rehabilitation services from the Norwegian Patient Register (NPR), the Municipal patient and user register (KPR), and the Norwegian Control and Payment of Health Reimbursements Database (KUHR) were used.

**Results:**

A total of 601 patients were included, 24% with moderate and 76% with severe injuries. The overall mean total health care cost per patient in the first year after traumatic injury was 846,877 (SD 1,042,649) Norwegian Kroner (NOK). The mean total cost of rehabilitation per patient was 251,487 (SD 317,050) NOK. Most costs were attributable to secondary care in the first six months post-injury. Severely injured patients had a higher health care utilization and average cost compared to those with moderate injury. Injury severity factors were the most prominent cost drivers, and number of injuries, severe head, spine, and extremity injuries were significantly associated with higher costs during the first year following trauma.

**Conclusions:**

The findings give a holistic insight into health care utilization and costs for patients across all ages with complex needs following trauma and can contribute to the planning and provision of services for this patient group.

## Background

Traumatic injuries, defined as a physical injury with a sudden onset, are a cause of disability, impaired quality of life and years of life lost [1, 2]. In Norway, approximately 10 000 persons were admitted to the regional trauma centers in 2021, either with a severe or potentially severe traumatic injury, with falls and traffic accidents being the most common causes of injuries [3]. Improvements in trauma care have significantly increased survival rates, especially in high-income countries [1]. Although patterns of recovery vary greatly between individuals, functional limitations and ongoing disability are common in the first year after injury [4, 5]. Studies have demonstrated long-lasting physical problems including psychological distress [1, 4, 6], and thus, a significant proportion of those surviving trauma will need complex rehabilitation and long-term follow-ups.

Besides the impact of injury on individuals themselves and their families, traumatic injuries pose a substantial economic burden on society in terms of health care utilization and health care costs. A previously published systematic review on acute treatment costs related to trauma found higher costs for this patient group compared to patients with other diseases [7]. Much of the previous studies on costs following trauma have focused solely on in-hospital costs and costs associated with the acute treatment and early rehabilitation [8, 9]. Considering the often long-lasting physical and psychological sequela for those who survive a moderate to severe traumatic injury, it is highly relevant to also include long-term costs of trauma to improve organizing and distribution of health care.

Although studies have shown variations in overall costs on both short [7] and long-term [10], several key factors have been identified as predictors for higher costs. This includes age, injury severity, needing surgical interventions, hospital and intensive care, length of stay, and polytrauma [7]. A systematic review of cost-drivers in acute treatment of severe trauma in Europe showed that length of the hospital stay and needing surgical interventions were the main cost-drivers in the acute phase suggesting that persons with multiple injuries are more likely to be in need of comprehensive surgical interventions and longer intensive care [9]. Others have identified that having sustained a spine or head injury is associated with higher costs [8, 11].

Of sociodemographic factors, female gender, higher age, and having comorbidities have shown to be predictors of high medical costs following trauma [11]. A study of adult trauma patients with severe injuries that included both in-hospital and post-hospital costs up to 2-years post injury, demonstrated that costs increased with injury severity, lower health status, extremity injury and injuries in multiple body regions [8]. The authors highlighted the importance of assessing long-term post-hospital costs and production loss as these constitute approximately 50% of the total costs [8].

Norway has a well-established welfare system and health care is publicly funded. Hospital services (inpatient stays, outpatient visits and rehabilitation at hospitals) are funded through block grants and activity-based funding based on diagnose-related groups (DRGs). Specialist and primary health care services are funded through the national insurance by a combination of block grants and activity-based funding, in addition to patients’ co-payments. Home and community based care is funded by the municipality and co-payment.

When patients are discharged from hospital, the municipalities are responsible for providing necessary health services and rehabilitation. These services can include contacts with primary health professionals and allied health professionals, as well as social services, equipment, and vocational support. Rehabilitation services are prescribed by clinicians based on clinical impairments and can be provided as in-patient or outpatient services, as well as community-based services.

The physical and psychological consequences of trauma are well recognized, however, we have previously shown that only 20% of persons with moderate to severe trauma are referred directly to rehabilitation after the acute trauma care, while 43% were discharged to local hospitals and 35% were discharged home [12]. It is likely to believe that a significant proportion of health care and rehabilitation services are provided outside the hospital. This includes specialist, home- and community-based health and rehabilitation services, as well as social services. However, part of the post-hospital costs is often neglected in economic evaluations [8, 13].

The aim of this study was to describe service utilization and costs for health care services, rehabilitation services, and social support services in the periods 0–6 months and 7–12 months after moderate to severe traumatic injury, as well as total costs at one-year post-injury. Further, we explored health care costs and their associations with sociodemographic (age, gender, geographical location), clinical and injury-related variables. Because rehabilitation needs following trauma were a particular interest of this study, factors associated with rehabilitation costs were assessed separately in addition to the factors associated with the total costs.

## Methods

### Design & ethics

This cost study was part of a larger mixed-method longitudinal multi-center study with prospective follow-ups at 6 and 12 months post-injury. The two regional trauma centers in the study were Oslo University Hospital and the University Hospital of North-Norway. The Regional Committee for Medical and Health Research Ethics (approval number 31676) and the Norwegian Data Inspectorate (approval number 19/26515) approved the study. Informed consent was obtained from all participants.

## Study population and recruitment

Patients of all ages admitted directly or within 72 h to either Oslo University Hospital or University Hospital of North Norway, in 2020 with moderate to severe traumatic injury determined by a New Injury Severity Score (NISS) > 9 and with a minimum 2-days hospital stay were eligible. Non-Norwegian residents and patients unable to speak/read Norwegian were excluded from the study. The National Institute of Clinical Excellence (NICE) guidelines states that persons with an Injury Sverity Score (ISS) of 9 or greater should be assessed for rehabilitation needs [14]. However, in this study, we used the NISS as its calculation methodology enables a more adequate rating of the severity of a patient presenting multiple injuries and identifies a higher amount of major trauma [15]. The patients and/or family members received information about the study from a project assistant and were included upon discharge from the acute hospital stay. The injury severity scores (New Injury Severity Score and Injury Severity Score) were initially calculated by physiatrist who were certified AIS registrars and later validated according to the scores registered in the local trauma registries at Oslo University Hospital and University Hospital of North Norway. Out of 1929 identified patients, 1214 were excluded, 49 patients did not respond and 65 patients declined participation. In total, 601 patients were included in the study.

## Data collection and data sources

The present study is based on data registered at baseline and data obtained from three national health registers, namely the Norwegian Patient Register (NPR), the Municipal patient and user register (KPR), and the Norwegian Control and Payment of Health Reimbursements Database (KUHR).

## Data recorded at baseline

Sociodemographic and injury-related variables were collected at baseline from medical records and the patients/relatives by the physiatrists in the project. Sociodemographic data included age, gender, living arrangements, and the patients’ geographical home region. Geographical region was determined based on the Norwegian Centrality Index score (NCI). The NCI scores are categorized based on a municipal’s centrality in terms of service functions and workplaces [16]. Clinical variables included American Society of Anaesthesiologists (ASA) Physical Status Classification System, a classification system for patients’ pre-injury somatic co-morbidities [17] and pre-injury psychiatric or substance abuse condition. Injury-related variables included date of injury and cause of injury, trauma-related diagnoses codes according to the International Classification of Diseases, Tenth Revision (ICD-10 diagnosis from S00-T32), intoxication at time of injury, the Abbreviated Injury Scale (AIS) – body region [18]. The AIS is an internationally used scale to classify injury severity ranging from 1 (minor) to 6 (maximal). An AIS score equal to or above 3 is considered as a severe injury, and in the present study AIS scores were dichotomized into mild-moderate injury (AIS 1–2) and severe to maximal injury (AIS 3–6) [18]. Additionally, global injury severity according to the Injury Severity Score (ISS) and the NISS, surgical and non-surgical treatment, length of the acute hospital stay, and discharge place were recorded.

## Data registers of service utilization and costs

The NPR includes information about hospital services in Norway, including ICD-10 diagnosis, outpatient visits and inpatient admissions, and includes medical as well as financial information. For each visit and admission it is assigned a diagnosis-related group (DRG) weight that reflect the resource use relative to the average hospital patient. One DRG point had a unit cost equal to Norwegian Kroner (NOK) 45 808 in 2020 and when combined with the DRG weight cost per visit or admission can be estimated [19]. We derived information on all treatments received in the hospitals related to a trauma ICD-10 code [20].

The KPR includes information about persons who have applied for, are receiving or have received services from the municipal health care service. Data in KPR includes diagnosis, number of patients who have received or applied for services, and the amount of time (hours/days) the service is provided [21].

The KUHR register holds information on claims and patient co-payments for visits to a range of health services that are partially publicly financed. Those services include, for example, visits to General Practitioners (GPs), emergency rooms, specialist doctors, outpatient activities (laboratories, radiology and psychiatry), physical therapists, rehabilitation institutions (only co-payments). When a patient is in contact with health professionals, a claim is sent to the Health Economics Administration (HELFO). HELFO is located under the Norwegian Directorate of Health. The information stored in KUHR includes ICD-10/ICPC-2 diagnose code, what kind of treatment that has been provided and information about the patient’s co-payment [22]. The ICPC-2 diagnosis is an international classification system for contacts made in the municipal health care system.

We obtained information on all activities and services recorded in the registers during the period from 1st of January 2020 to 28th February 2022. Services not related to a trauma diagnosis according to ICD-10/ICPC-2 were excluded from the study. However, an exception was made for specialist practitioners who report claims to the KUHR, i.e., ear-nose-throat doctor, psychologist and ophthalmologist. Patients who have sustained traumatic injury are commonly referred to these specialists, however, the services may be registered under other diagnosis codes than trauma codes and excluding these services may lead to an underestimation of costs. Health care services and variables used to describe health care utilization and costs are reported in Table 1.

**Table 1:** Health care services and variables used to describe health care utilization and costs

Services	Units	Source
Secondary care		
Hospital admissions	Visits	NPR
Day treatments	Visits	NPR
Outpatient visits (Hospital)	Visits	NPR
Other costs^a^	Visits	KUHR
**Rehabilitation**		
Private rehabilitation institutions (Secondary care)	Days	NPR
Physical therapist	Visits	KUHR
Communal rehabilitation services (time limited stay) Functional assessment and rehabilitation	Days	KPR/KUHR
**Primary care**		
General practitioner	Visits	KUHR
Emergency room	Visits	KUHR
**Specialists**		
Ophthalmologist	Visits	KUHR
Ear, nose, throat (ENT)	Visits	KUHR
Internal medicine	Visits	KUHR
Physicians	Visits	KUHR
Surgeon	Visits	KUHR
Psychologist	Visits	KUHR
Psychomotor physiotherapist	Visits	KUHR
Neurologist		
**Home- and community-based care**		
Short-term institution	Days	KPR/KUHR
Long-term institution	Days	KPR/KUHR
Day care service	Days	KPR/KUHR
Home-based services (practical assistant, nurse, physical therapist, occupational therapist, psychiatric nurse, health care worker)	Hours	KPR/KUHR
Social support (support contact)	Hours	KPR

^a^Other costs not captured in DRG: such as laboratory, radiology and patient co-payment

### Cost calculations

Service utilization and costs were calculated based on two six-month periods: from 0 to 6 months post-injury and 7–12 months post-injury, as well as total at 12-months post-injury. Health care costs were categorized as *primary care* (i.e., GPs and emergency room), *specialist outside the hospital* (i.e., ear, nose, throat, ophthalmologist, and psychologist), and *secondary care* (hospital admissions, outpatient visits, and day care services), *rehabilitation* (rehabilitation units, private institutions, communal rehabilitation institutions, and physical therapist), *home and community-based care* (short, and long term institutions, day care services, practical assistant, nurse, occupational therapist, physical therapist at home, and social support), see Table 1. Rehabilitation costs were further separated into complex rehabilitation and usual rehabilitation, which patients receive either in hospital or in private institutions. Complex rehabilitation is characterized by individually tailored and goal-oriented services provided by the multidisciplinary team (at least six health professionals) led by a medical doctor specialized in physical medicine and rehabilitation over a time of at least five days, with a minimum of one overnight stay at the institution where the patients receive functional training, training in compensatory techniques and adaptation of aids/environment. For usual rehabilitation the required number of team professionals is four (Norwegian Directorate of Health, 2023).

For secondary care (NPR) the costs were estimated as 100% of DRG costs. For primary care it has been stated in a report from the Norwegian Health Directory that the reimbursement claims and patients’ co-payment reported in KUHR corresponds to approximately 30% of the total costs of treatment. Thus, we summarized the total reimbursements and patient co-payments and multiplied them by 2, as was done in a previous cost study [23]. The average costs of primary health care services, rehabilitation institutions, and specialists were based on reimbursement claims, patient co-payments, and out-of-pocket payments for the respective visits for patients using these services. For home- and community-based care, days in an institution were multiplied with corrected gross operating expenses published by Statistics Norway (SSB) as part of the Municipality-State-Reporting [24]. Table 2 present an overview of cost components and calculations.

**Table 2:** Cost calculations. All costs are presented in Norwegian kroner (NOK)

Health care costs	Category	Source	Calculation
Secondary care costs	Inpatient stay	NPR	DRG weight*unit cost
	Day treatment	NPR	DRG weight*unit cost
	Outpatients stay	NPR	DRG weight*unit cost
	Complex rehabilitation	NPR	DRG (462A) weight*unit cost
	Usual rehabilitation	NPR	DRG (462B) weight*unit cost
	Other costs not captured by DRG	KUHR	(Claim + co-payment) *2
Primary care cost	Visits to GPs, emergency room, Physical therapists, etc.	KUHR	(Claim + co-payment) *2
Home- and community- based care	Long-term and short-term institutions, home care	KPR	Time in institution/home*Unit cost
Total costs	All categories above	-	Sum of total secondary, primary, and home- and community-based care costs

In 2021, €1 = NOK 10

### Statistical analysis

#### Descriptive analyses

Sociodemographic, socioeconomic, and injury-related variables are presented by means and standard deviations (SDs), medians and interquartile ranges (IQRs), or frequency distribution across the complete sample, as well as across moderate and severe injury severity as classified by NISS. Health care use and cost were provided for the first year following injury date and are reported as average cost/number of visits per patient across the periods 0–6 months post-injury, and 7–12 months post-injury, and 12 months post-injury.

To explore the associations between sociodemographic and clinical variables and costs, we conducted multivariate linear regression analyses on the total costs and specifically on the costs of rehabilitation. The variables of interest were chosen based on clinical experience and previous research and included gender (male/female), age (children < 18 years / adults ≥ 18 years), geographic region (central/less central), living conditions (living alone/living with others), pre-injury ASA score (systemic disease ASA 2-4/healthy ASA 1), pre-injury psychiatric condition and/or substance abuse (no/yes), number of injuries, severe head injury (no/yes), severe spine injury (no/yes), severe abdominal injury (no/yes), severe thorax injury (no/yes), severe upper/lower extremity injury (no/yes). Generalized linear models (GLM) were conducted and tested using different link distributions (e.g., Gamma, Gaussian, and inverse Gaussian distributions and identity, log and power links). The models with the lowest Akaike information criterion (AIC) and Bayesian information criterion (BIC) were selected, and the results are presented in the main text. To interpret results according to the variables original scale, the results of the regression models are presented as the average marginal effects (AME) [25]. The significance level was set to *P* < 0.05, and all analyses were conducted using STATA SE, version 18.

## Results

### Description of the study sample

A total of 601 patients (75% males) were included in the study. Mean (SD) age was 47 (21.2) years. The highest proportion of patients were between the age of 55 and 74 years. Most patients were cohabiting or married, and 56% lived in central areas as classified by the Centrality Index Score. Approximately half (54%) of the patients were classified as healthy prior to the trauma according to their American Society of Anaesthesiologists (ASA) physical status classification. Falls, followed by transportation accidents, were the most common causes of injury and 76% of the patients had sustained a severe traumatic injury as classified by a NISS > 15. Approximately, 42% had a severe head injury (AIS head ≥ 3), while 33% had a severe thorax injury. Mean length of acute hospital stay was nine days for the whole sample, and 22% had been discharged directly from the acute care to rehabilitation units. A higher proportion of those with severe injury (27%) were directly referred to rehabilitation compared to those with a moderate injury (8%). During the study period, 19 (3%) patients died, but we have no information about the cause of deaths. Table 3 displays the sociodemographic and injury-related characteristics of all included patients.

**Table 3:** Descriptive sociodemographic, clinical and injury-related characteristics of the study population of patients with moderate to severe traumatic injury (*n* = 601)

Variables	All (*n* = 601)	Moderate (NISS ≤ 15) (*n* = 144)	Severe (NISS > 15) (*n* = 457)
	N (%)	N (%)	N (%)
**Age (years)**			
0–17	63 (10.5)	17 (11.8)	46 (10.1)
18–24	48 (8.0)	13 (9.0)	35 (7.7)
25–34	75 (12.5)	23 (16.0)	52 (11.4)
35–44	75 (12.5)	16 (11.1)	59 (12.9)
45–54	86 (14.3)	23 (16.0)	63 (13.8)
55–64	103 (17.1)	17 (11.8)	86 (18.8)
65–74	101 (16.8)	20 (13.9)	81 (17.7)
75+	50 (8.3)	15 (10.4)	35 (7.7)
**Gender**			
Male	451 (75.0)	107 (74.3)	344 (75.3)
**Marital status**			
Single	181 (30.1)	47 (32.6)	134 (29.3)
Live with parents	75 (12.5)	21 (14.6)	54 (11.8)
Cohabitating/married	309 (51.4)	67 (46.5)	242 (53.0)
Separated/divorced	17 (2.8)	3 (2.1)	14 (3.1)
Widowed	17 (2.8)	5 (3.5)	12 (2.6)
Missing	2 (0.3)	1 (0.7)	1 (0.2)
**Geographical index**			
Central (Centrality Index 1–2)	337 (56.1)	76 (52.8)	240 (52.5)
Less central (Centrality Index 3–6)	264 (43.9)	68 (47.2)	217 (47.5)
**Employment status at time of injury**			
Employed	300 (49.9)	70 (48.6)	230 (50.3)
Unemployed	114 (19.0)	24 (16.7)	90 (19.7)
Not applicable (Child)	60 (10.0)	15 (10.4)	45 (9.9)
Retired	113 (18.8)	29 (20.1)	84 (18.4)
Missing	14 (2.3)	6 (4.2)	8 (1.8)
**ASA score**			
Healthy (ASA 1)	327 (54.4)	90 (62.5)	237 (51.9)
Systemic disease (ASA 2–6)	274 (45.6)	54 (37.5)	220 (48.1)
***Injury-related variables***			
**Cause of injury**			
Transport accident	227 (37.8)	55 (38.2)	172 (37.6)
Fall	256 (42.6)	57 (39.6)	199 (43.4)
Violence	18 (3.0)	4 (2.8)	14 (3.1)
Others	100 (16.4)	28 (19.4)	72 (15.8)
**AIS ≥ 3**			
Head ≥ 3	250 (41.6)	19 (13.2)	231 (50.6)
Spine ≥ 3	80 (13.3)	18 (12.5)	62 (13.6)
Thorax ≥ 3	197 (32.8)	14 (9.7)	183 (40.0)
Abdomen ≥ 3	73 (12.2)	11 (7.6)	62 (13.6)
Upper/lower extremities ≥ 3	101 (16.8)	19 (13.2)	82 (17.9)
Injury Severity Score (ISS) Mean (SD)	18.5 (10.0)	9.4 (SD 2.2)	21.4 (SD 9.4)
Length of hospital stay (days)	8.6 (SD 9.0)	6.08 (SD 6.3)	9.4 (SD 9.6)
**Discharge place**			
Home	211 (35.1)	86 (59.7)	125 (27.4)
Local hospital	237 (39.4)	39 (27.1)	198 (43.3)
Rehabilitation	134 (22.3)	12 (8.3)	122 (26.7)
Nursing home/other	19 (3.2)	7 (4.9)	12 (2.6)

*ASA The American Society of Anesthesiologists classification of physiological status*

### Health care utilizations & descriptions of costs

Table 4 displays an overview of service utilization related to the first year following traumatic injury. At 12 months post-injury, the patients had on average of 3.5 inpatient admissions. Looking at the first six months, the patients had a mean (SD) of 3.4 (1.7) hospital admissions, while a mean (SD) of 1.8 (0.9) hospital admissions from 7 to 12 months after injury. At 12-month following the injury, patients with severe injuries had a higher average number of inpatient hospitalizations, outpatient visits and other secondary care services than patients with moderate injuries. Within primary care, then mean number of GP visits were 16.3 times per patient at 12-month after the injury, where patients with a severe injury on average visited the GP 5 more times relative to patients with moderate injuries. In patients receiving specialist services, the mean number of specialist visits at one year after the injury were broadly similar in the moderate and severe groups (3.1 visits vs. 3.4 visits), and the average number of specialist visits remained stable across the 0–6- and 7–12-month time periods (2.1 vs. 2.6).

**Table 4:** Average number of visits in different level of care for the study population by time since injury and severity of injury

Time since injury		0–6 months			7–12 months			12 months		
		**All**	**Moderate (NISS ≤ 15)**	**Severe (NISS > 15)**	**All**	**Moderate (NISS ≤ 15)**	**Severe (NISS > 15)**	**All**	**Moderate (NISS ≤ 15)**	**Severe (NISS > 15)**
Level of care	Category	Mean (SD)			Mean (SD)			Mean (SD)		
**Secondary care**	Inpatient admissions^a^	3.39 (1.65)	2.91 (1.52)	3.54 (1.66)	1.79 (0.92)	2.29 (1.25)	1.62 (0.64)	3.48 (1.79)	3.03 (1.84)	3.62 (1.76)
	Day treatments	1.40 (0.69)	1.38 (0.81)	1.41 (0.66)	1.31 (1.01)	1.33 (0.58)	1.31 (1.11)	1.43 (0.74)	1.4 (0.81)	1.44 (0.73)
	Outpatient admissions	3.27 (3.55)	2.99 (2.64)	3.35 (3.78)	2.51 (3.09)	1.51 (1.10)	2.79 (3.39)	4.05 (4.78)	3.45 (2.94)	4.22 (5.18)
	Other secondary care services^b^	8.96 (6.65)	8.83 (5.94)	9.00 (6.86)	6.12 (6.62)	4.88 (5.88)	6.48 (6.79)	13.71 (11.10)	12.36 (9.98)	14.13 (11.41)
**Primary care**	General Practitioner (visits)	9.26 (9.25)	7.69 (6.75)	9.74 (9.84)	8.48 (8.72)	6.40 (5.40)	9.11 (9.43)	16.28 (15.60)	13.08 (10.75)	17.25 (16.69)
	Emergency room (visits)	1.99 (1.84)	1.82 (1.01)	2.05 (2.06)	2.36 (3.32)	2.10 (1.93)	2.44 (3.63)	2.77 (3.76)	2.48 (2.21)	2.86 (4.14)
**Specialist care**^c^	Specialists (visits)	2.07 (2.11)	2.03 (1.94)	2.09 (2.20)	2.60 (3.45)	2.04 (1.93)	2.81 (3.87)	3.29 (4.19)	3.1 (3.44)	3.37 (4.46)
**Rehabilitation**	Inpatient rehabilitation stays in days	26.71 (11.97)	25.5 (7.78)	26.81 (12.34)	38.14 (15.15)	. (.)	38.14 (15.15)	29.85 (14.02)	25.5 (7.78)	30.13 (14.35)
	Day treatment in private rehabilitation institutions	25 (.)	. (.)	25 (.)	8 (.)	. (.)	8 (.)	16.5 (12.02)	. (.)	16.5 (12.02)
**Home- and community- based care**	Short-term stay^d^ in days	32.26 (34.23)	21.43 (28.54)	33.91 (34.99)	39 (40.12)	. (.)	39 (40.12)	48.42 (59.40)	21.43 (28.54)	52.2 (61.75)
	Long-term stay^e^ in days	60 (30)	60 (.)	60 (32.07)	110 (69.28)	. (.)	110 (69.28)	178 (124)	240 (.)	173 (128)
	Home-based services^f^ in hours per week	5.90 (21.77)	1.23 (1.98)	7.02 (24.09)	7.02 (17.34)	2.49 (3.91)	7.77 (18.59)	7.36 (25.81)	1.78 (2.99)	8.56 (28.31)

^a^Excluding inpatient rehabilitation admissions.

^b^Other services not captured in DRG, such as laboratory, radiology and patient co-payment.

^c^This includes ophthalmologist, Ear, nose and throat (ENT) specialist, Internal medicine specialist, surgeon, psychologist, neuropsychologist, psychomotor physiotherapist, and neurologist.

^d^This includes care in institution/outside institution, time limited stay (assessment/treatment) and time limited stay (others)

^e^This refers to long-term stays in institutions

^f﻿^Includes health services at home and practical assistance

On average, patients in the moderate and in the severe group spent similar number of days at inpatient rehabilitation stays in the first six months post-injury, but none of the patients in the moderate group received this service in the second 7–12-month period. None of the patients in the moderate group received day treatment in private rehabilitation institutions.

One year after injury, patients spent on average 48 days in a short-term stay in institutions provided by home and community services, and 178 days in long-term stays. At 12 months, patients with severe injuries had an average 31 days longer short-term stay than patients with moderate injuries (52 days vs. 21 days). During the same period, the severely injured patients also received an average of 7 h more home-based services per week than the moderately injured patients (8.6 vs. 1.8 h). Patients in the severly injured group had a higher health care utilization within all categories at 12-months post-injury, except for long-term stay in institution. However, the numbers are reported per patient who received the service and thus, the number of days or visits may be high for the few in the moderately injured group who received this service.

### Description of costs

Table 5 displays costs categories and total costs according to time since injury and injury severity, as well as the total costs related to traumatic injuries the first-year post-injury.

**Table 5:** Average costs per patient by time since injury and severity of injury

Time since injury		0–6 months			7–12 months			12 months		
		**All**	**Moderate (NISS ≤ 15)**	**Severe (NISS > 15)**	**All**	**Moderate (NISS ≤ 15)**	**Severe (NISS > 15)**	**All**	**Moderate (NISS ≤ 15)**	**Severe (NISS > 15)**
Level of care	Category	Mean (SD) cost			Mean (SD) cost			Mean (SD) cost		
**Secondary care**	Inpatient admissions^a^	633,452 (802,920)	282,076 (249,440)	741,506 (880,108)	150,311 (192,441)	240,416 (261,367)	120,276 (160,352)	640,784 (810,819)	294,542 (273,493)	747,259 (888,180)
	Day treatments	8,844 (14,977)	8538 (6108)	8934 (16,725)	15,125 (12,331)	16,881 (10,401)	14,720 (13,080)	9402 (15,265)	9017 (7057)	9515 (16,956)
	Outpatient admissions	7,438 (8198)	6257 (5901)	7797 (8754)	6471 (8668)	3639 (3823)	7242 (9436)	9494 (12,103)	7382 (7172)	10,114 (13,149)
	Other secondary care services^b^	6,559 (6032)	6674 (6040)	6522 (6036)	5692 (8360)	4437 (6242)	6053 (8850)	11,008 (11,558)	9898 (10,030)	11,358 (11,988)
	Total	621,948 (797,862)	279,173 (255,084)	730,192 (876,741)	17,608 (61,109)	22,187 (87,801)	16,280 (50,908)	635,653 (809,299)	295,351 (280,150)	743,116 (888,459)
**Primary care**	General Practitioner	5769 (6617)	5181 (5751)	5947 (6855)	5797 (6629)	5126 (8648)	6003 (5869)	10,595 (11,252)	9534 (12,690)	10,916 (10,775)
	Emergency room	2177 (2153)	1887 (1746)	2281 (2277)	1478 (2862)	976 (1302)	1624 (3162)	2363 (3482)	1915 (1967)	2507 (3838)
	Total	6522 (7224)	5953 (5956)	6695 (7563)	6181 (7234)	5222 (8478)	6483 (6779)	11,748 (12,272)	10,202 (12,591)	12,233 (12,144)
**Specialist care**^c^	Specialists (KUHR)	5334 (5471)	5724 (6287)	5145 (5069)	6603 (9972)	4816 (4598)	7279 (11,317)	8418 (10,877)	8094 (8585)	8543 (11,675)
**Rehabilitation**	Inpatient rehabilitation^d^ (NPR)	350,079 (299,308)	226,792 (273,800)	361,548 (299,728)	58,808 (70,434)	53,960 (18,220)	59,248 (73,464)	363,162 (309,072))	226,148 (268,322)	376,705 (310,216)
	Inpatient complex rehabilitation (NPR)^e^	375,487 (297,331)	255,787 (281,521)	386,439 (297,203)	63,925 (74,531)	51,391 (24,409)	64,915 (77,244)	388,852 (307,169)	249,012 (276,329)	402,562 (307,428)
	Inpatient usual rehabilitation (NPR)^f^	87,932 (67,784)	28,031 (.)	89,191 (68,007)	20,183 (20,505)	61,669 (.)	15,573 (15,297)	72,117 (66,527)	44,850 ( 23,785)	73,416 ( 67,750)
	Inpatient stays in private rehabilitation institutions	106,857 (47,875)	102,000 (31,113)	107,231 (49,342)	152,571 (60,594)	. (.)	152,571 (60,594)	119,412 (56,062)	102,000 (31,113)	120,500 (57,391)
	Day treatment in private rehabilitation institutions	50,000 (.)	. (.)	50,000 (.)	16,000 (.)	. (.)	16,000 (.)	33,000 (24,042)	. (.)	33,000 (24,042)
	Physical therapists (Primary care)^g^	14,239 (13,524)	16,438 (16,274)	13,643 (12,669)	21,011 (19,347)	23,684 (21,976)	20,396 (18,712)	28,319 (28,972)	29,927 (34,750)	27,882 (27,280)
	Short stay in communal rehabilitation institutions: (KPR)^h^	139,868 (147,229)	71,010 (64,823)	148,696 (152,913)	138,075 (138,357)	118,350 (.)	142,020 (154,310)	159,515 (167,776)	78,900 (61,116)	171,608 (175,644)
	Total	240,048 (292,260)	84,956 (172,913)	273,739 (302,075)	39,889 (57,930)	30,263 (28,413)	41,994 (62,415)	251,487 (317,050)	95,905 (176,972)	286,672 (331,061)
**Home- and community- based care**	Short-term stay^i^	127,282 (135,038)	84,536 (112,573)	133,787 (138,038)	153,855 (158,292)	. (.)	153,855 (158,292)	191,021 (234,315)	84,536 (112,573)	205,929 (243,615)
	Long-term stay^j^	236,700 (118,350)	236,700 (.)	236,700 (126,522)	433,950 (273,318)	. (.)	433,950 (273,318)	703,525 (487,994)	946,800 (.)	681,409 (505,466)
	Home-based services^k^	18,621 (58,057)	6599 (14,145)	21,492 (63,967)	18,331 (40,649)	8739 (12,463)	19,930 (43,513)	66,639 (271,889)	14,404 (33,869)	77,932 (298,415)
	Total	65,395 (126,040)	29,704 (92,816)	73,836 (131,533)	64,459 (136,490)	8739 (12,463)	72,107 (143,961)	158,209 (387,017)	30,077 (74,782)	179,914 (412,913)
**Total costs**	Total	778,992 (948,702)	327,380 (320,105)	921,606 (1,033318)	44,849 (92,662)	32,753 (85,817)	48,727 (94,522)	846,877 (1,042649)	357,524 (325,560)	999,333 (1,137105)

In 2021, €1 = NOK 10

^a^Excluding costs of inpatient rehabilitation admissions.

^b^This includes costs of other services not captured in DRG, such as laboratory, radiology and patient co-payment.

^c^This includes costs associated with ophthalmologist, Ear, Nose and Throat doctor (ENT) specialist, Internal medicine specialist, surgeon, psychologist, neuropsychologist, psychomotor physiotherapist, and neurologist.

^d^This includes DRGs: 462A, 462B, and 462C.

^e^Includes DRG=462A complex rehabilitation.

^f^This includes DRG=462B usual rehabilitation.

^g^This includes costs associated with various services such as simple patient contact by letter, phone call or electronic communication, examinations, treatments, group treatments, guided exercises, necessary conversation with next of kin or guardian.

^h^This includes costs associated with time-limited stay in rehabilitation institutions.

^i^This includes costs that occurred during short-term stays in institutions, care outside institution, time limited stay for medical examination and treatment, and other short-term stay.

^j^This includes costs that occurred during long-term stays in institution.

^k^This includes costs associated with health services at home (home-based health services: using monthly wage of health professionals in year 2021) and practical assistance (using monthly wage of cleaners/nursing staff in year 2021).

The overall mean total cost per patient in the first year after traumatic injury was 846,877 NOK, and the total cost per patient in the first 6 months after injury was 16-times higher than the total 7–12 months average cost (778,992 vs. 44,849 NOK). In the first year after injury, the average total cost was almost three times as high for those with severe injury (999,333) compared to those with moderate injury severity (357,524). The largest cost component during the first year after injury was secondary care services, with an average total cost of 635,653 NOK. In the secondary care category, the majority of costs can be attributed to inpatient admissions. Comparing the average total cost of secondary care in the 0–6 months and 7–12 months after injury, the average cost in the first 6 months after injury was substantially higher: the average total cost of secondary care for 0–6 months was 621,948NOK, while the average total cost of secondary care for 7–12 months after injury was 17,608 NOK.

The second largest cost component 12 months after injury was rehabilitation services with an average total cost of 251,487 NOK followed by home- and community-based care services (158,209). The average rehabilitation cost for the severely injured group was three times as high compared to those with moderate injury (286,672 vs. 95,905). There are no moderately injured patients who had inpatient stays or received day treatment in private institutions within 7–12 months after injury, therefore costs in these two categories were zero. The average cost per patient for complex rehabilitation was five times the average cost per patient for usual rehabilitation (388,852 vs. 72,117).

For home- and community-based services, the average total cost for patients with severe injuries (179,914 NOK) was almost 5-times higher than for patients with moderate injuries (30,077 NOK). The average total costs in home- and community-based care were stable across the two follow-up periods. Although average costs per patient were higher in most cost categories for severely injured patients, costs per patient in patients with moderate injury were higher for physical therapy during the first six months after injury. Patients with moderate injury had a higher average cost of physical therapy compared to patients with severe injury (16,438 vs. 13,643).

### Associations/predictors between sociodemographic, clinical, injury-related characteristics and total costs

Table 6 shows the results from the regression model of total costs at 12 months post-injury related to moderate and severe traumatic injuries. Being classified as healthy according to the ASA score was significantly associated with lower total costs (−212,121 NOK, *p* = 0.017). Of the injury-related variables, increase in the number of injuries was significantly associated with higher cost (101,476 NOK, *p* < 0.001). Further, having a severe (classified as an AIS ≥ 3) head, spine, and upper/lower extremity injury was significantly associated with higher total cost at 12-months post-injury. Patients with a severe head injury had 337,252 NOK higher total costs (*p* = 0.002), for severe spine injury the cost was 675,126 NOK higher (*p* = 0.002), and for severe upper/lower extremity injury the cost was 441,114 NOK higher (*p* = 0.004) compared to those with an AIS < 3.

**Table 6:** GLM model of total costs (NOK) with gamma distribution with identity link. Number of observations *n* = 559

Variable	Category	Margins	Robust st. error	*P*-value	95% CI	
Age group	Children^α^ vs. adult	100,923	137,122	0.462	−167,832	369,677
Gender	Male^α^ vs. female	68,713	84,248	0.415	−96,409	233,835
Centrality Index	Central ^α^ vs. less central	68,713	84,248	0.415	−96,409	233,835
Living arrangement	Living alone^α^ vs. cohabitating	−48,618	89,345	0.586	−223,731	126,495
**Pre-injury health status ASA**	Systemic disease^α^ vs. healthy	**−212**,**121**	**88**,**909**	**0.017**	−386,379	−37,862
Pre-injury psychiatric/substance abuse	No ^α^ vs. yes	−46,909	−46,909	0.648	−248,142	154,324
**Number of injuries**	Continuous	**101**,**476**	**17**,**155**	**< 0.001**	67,852	135,100
**Severe head injury (AIS ≥ 3)**	No ^α^ vs. yes	**337**,**252**	**109**,**939**	**0.002**	121,776	552,729
**Severe spine injury (AIS ≥ 3)**	No ^α^ vs. yes	**675**,**126**	**214**,**855**	**0.002**	254,017	1,096,234
Severe abdominal injury (AIS ≥ 3)	No ^α^ vs. yes	181,355	154,949	0.242	−122,340	485,050
Severe thorax injury (AIS ≥ 3)	No ^α^ vs. yes	−105,442	101,936	0.301	−305,233	94,350
**Severe upper/lower extremity injury (AIS ≥ 3)**	No ^α^ vs. yes	**441**,**114**	**154**,**718**	**0.004**	137,871	744,356

α = reference category; AIC: 28.79283; BIC: −2998.523; In 2021, €1 = NOK 10

Table 7 shows the result from the regression model of factors associated with total rehabilitation costs at 12 months post-injury. As for the total cost, an increase in the number of injuries was significantly associated with higher rehabilitation costs (76,461 NOK, *p* = 0.023). Patients with severe head injury (AIS ≥ 3) had 296,923 NOK higher rehabilitation costs. Patients with severe upper/lower extremity injury had significantly higher rehabilitation costs (442,930 NOK, *p* = 0.017) at 12 months post-injury. Severe abdominal injury (−248,156NOK, *p* = 0.011) and severe thorax injury (−293,636 NOK, *p* = 0.050) were significantly associated with lower rehabilitation costs. This was somewhat unexpected, and we did additional analyses where we found that only 15% of patients with severe abdominal injury (AIS ≥ 3) also had severe head injury compared to 45% in the less severe group (AIS < 3). Further, only 9% of the patients with severe abdominal injury had severe extremity injury compared to 17% in the less severe group. Similar trend was found regarding thorax injuries, where 30% of those with severe thorax injury had severe head injury compared to 48% in the less severe group.

**Table 7:** GLM model of total rehabilitation costs (NOK) with gamma distribution with identity link. Number of observations *n* = 559

Variable	Category	Margins	Robust st. error	*P*-value	95% CI	
Age group	Children^α^ vs. adult	32,390	97,000	0.738	−157,726	222,506
Gender	Male^α^ vs. female	126,474	95,214	0.184	−60,142	313,090
	Centrality Index Central ^α^ vs. less central	−85,596	66,236	0.196	−215,415	44,224
Living arrangement	Living alone^α^ vs. cohabitating	−42,557	65,373	0.515	−170,686	85,571
Pre-injury health status ASA	Systemic disease^α^ vs. healthy	−41,004	64,977	0.528	−168,357	86,349
Pre-injury psychiatric/substance abuse	No ^α^ vs. yes	38,060	74,578	0.610	−108,110	184,231
**Number of injuries**	Continuous	**76**,**461**	**33**,**564**	**0.023**	10,676	142,246
**Severe head injury (AIS ≥ 3)**	No ^α^ vs. yes	**296**,**923**	**106**,**114**	**0.005**	88,944	504,902
Severe spine injury (AIS ≥ 3)	No ^α^ vs. yes	1,618,329	871,375	0.063	−89,534	3,326,192
**Severe abdominal injury (AIS ≥ 3)**	No ^α^ vs. yes	**−248**,**156**	**97**,**359**	**0.011**	−438,975	−57,337
**Severe thorax injury (AIS ≥ 3)**	No ^α^ vs. yes	**−293**,**636**	**149**,**764**	**0.050**	−587,168	−105
**Severe upper/lower extremity injury (AIS ≥ 3)**	No ^α^ vs. yes	**442**,**930**	**185**,**795**	**0.017**	78,780	807,081

α = reference category; AIC: 24.88567; BIC: −2640.436; In 2021, €1 = NOK 10

## Discussion

This study provides a description of health care utilization and costs associated with health care services, rehabilitation services, and social support services in a cohort of persons who had sustained moderate or severe traumatic injury. In addition, we assessed the association between sociodemographic, clinical and injury-related factors and total costs and rehabilitation costs. Overall, most costs were attributable to secondary care in the first six months post-injury, and there was a decline in the average visits per patient of formal health care services in the secondary care category from 0 to 6 months to 7–12 months after injury. The mean number of visits per patient within the primary care category, including visits to GP and specialists, remained stable throughout the first-year post-injury. Patients with a moderate injury (NISS ≤ 15) had on average a lower number of visits/days per patient in rehabilitation and home- and community-based care. As expected, this trend was evident in the period 7–12 months post-injury, indicating that patients with moderate injuries received less rehabilitation and community-based services in the first year compared to severly injured.

### Overall costs

Patients with severe traumatic injury (NISS > 15) had a mean total cost per patient almost three times as high as the mean total cost per patient in the moderately injured group. In total, 75% of the total costs were attributable to secondary care services, mainly inpatient admissions, where most of the costs were related to the first six months after injury. Our findings are in line with an English study on 668 persons (aged 16–70 years) with traumatic injury demonstrating that 87% of the mean costs in the first year after injury were related to secondary care services [26]. Further, the total average cost per patient for secondary care decreased with 97% from 0 to 6 months to 7–12 months post-injury, which indicate that most of the secondary care services are provided in the acute and sub-acute phases after injury.

Patients classified as severely injured (NISS > 15) had 2.5 times higher secondary care costs at 12 months compared to moderately injured patients (NISS ≤ 15). Severity of injury as a cost driver has been demonstrated in other studies as well. In a study of medical costs and productivity loss from the Netherlands, patients with severe injuries, determined by a an ISS ≥ 25, had significantly higher mean in-hospital care costs compared to patients with ISS between 16 and 24 [8]. However, the total mean in-hospital care costs per patient were lower in the study by van der Vlegel et al. compared to our results. One explanation for this, as pointed out by the authors themselves, might be that they did not include costs related to surgical procedures [8].

Another study investigating prognostic factors for medical and productivity costs up to two years after trauma, found a mean medical cost per patient of 9,710 Euros including both in-hospital and post-hospital costs [11]. Compared to these studies, the health care services provided to trauma patients are more costly in Norway. However, the Norwegian health care system is mainly publicly funded and has been considered among the most expensive in Europe [27]. In 2022, Norway spent more money on health care per individual than any of the other Nordic countries [28]. It should also be considered that differences in cost might partly be explained by the use of different costing methods employed in studies. In a systematic review of the average cost per patient for acute trauma treatment in high-income countries, both large variance in costs between countries and differences in costing methods used were evident [7].

The present study encompassed patients of all ages. Gender and age group (dichotomized into children vs. adults) showed no significant association with the mean total costs in our study. In the study by Munter et al., female gender and older age (≥ 75 years) were significantly associated with higher mean medical cost [11]. However, the study by Munter et et al. did not include children, and the mean age was higher than in our study and included more females. In our study, the majority (75%) of the patients were males, which is in line with the trend in Norway where men dominate the trauma statistics in almost all age groups apart from the oldest age groups where woman dominate [3].

Being classified as a normal healthy person according to the ASA physical status score at time of injury was significantly associated with lower total costs at 12-months post injury. We have previously shown that being healthy at time of injury was a significant predictor for better functional outcomes six months post-injury when assessed with Glasgow Outcome Scale – Extended [29]. Patients who recover better are less likely to need of formal health care and rehabilitation services, and thus, cost less. This underlines the importance of implementing preventive strategies to support and improve public health, which can reduce the economic burden due to health care service provision to persons who have sustained a traumatic injury.

The number of injuries was associated with higher mean total costs at one-year post-injury, and an increase in number of injuries was associated with 101,476 NOK higher costs. Higher mean costs in patients with multiple injuries have also been demonstrated in several other studies on the trauma population [7, 8, 26]. Furthermore, having a severe injury (AIS ≥ 3) in the head, spine, and upper/lower extremities were significantly associated with higher mean total costs indicating that severe injuries in these body regions have consequences that require more health care services. Injuries in these body regions have also been associated with higher costs in other studies [7, 11].

As previously mentioned, most cost studies on the trauma population have focused solely on in-hospital care costs, while costs related to home- and community-based services and rehabilitation services are often neglected [8]. We have previously shown that one half of the trauma survivors had a persistent disability at 12 months post-injury [29] and that 46% reported unmet needs for community-based rehabilitation services at 12-months post-injury [30]. Home- and community-based services were the third largest cost component in this study, with a mean cost per patient of 158,209 NOK. The mean cost per patient for home and community-based services was almost 6-times as high for those with severe injury compared to those with a moderate injury. Based on the recommendation of coordinated and complex rehabilitation for this patient group, and [31] the previous findings of a significant proportion of people having unmet rehabilitation needs, it was of interests to look specifically at costs related to rehabilitation services.

### Rehabilitation costs

Rehabilitation is a core component of the service provision after trauma due to the increase in survival rates, and provision of complex rehabilitation is stated in the guidelines of the Norwegian National Trauma Plan [31]. The mean total cost of rehabilitation per patient was 251,487 NOK (SD 317,050). The mean cost per patient was highest in the first six months after injury and decreased by 83% from the 0–6 months period to the 7–12 months post injury period.

The total rehabilitation cost was higher for patients with severe injury compared to patients with moderate injury across all time periods. The mean cost per patient for complex rehabilitation was five-times higher than the mean cost per patient for usual rehabilitation. Severely injured patients had almost twice as high mean cost for complex and usual rehabilitation compared to moderately injured patients. The average number of days spent in rehabilitation institutions was a little less than a month for both groups in the 0–6 months period. While the average number of days/visits in rehabilitation institutions increased to almost 40 days for the severe group in the second period, none of the patients in the moderate group were registered with stays or day treatment at rehabilitation institutions in the 7–12 months period.

Contrary, patients with moderate injury had a higher mean cost per patient related to physiotherapy compared to patients with severe injury. In Norway, the municipalities have to statutorily guarantee access to publicly funded physiotherapy [32]. Physiotherapy is thus a service which is accessible to many, and it might be likely that patients with moderate injuries who were not referred to specialized rehabilitation or rehabilitation institutions sought physiotherapy to support rehabilitation. Other studies have demonstrated that physiotherapy is the most frequently delivered service to persons with traumatic brain injury [33, 34].

None of the sociodemographic factors were significant drivers for rehabilitation costs. We have previously shown that living less centrally significantly increased the odds of having unmet needs for community-based rehabilitation services at 6 months-post injury [35]. Thus, one could expect that living less centrally would also be a significant factor associated with lower rehabilitation costs. In a systematic review on the influence of geographical factors on outcomes following trauma, it was found that patients located in rural areas often had a longer hospital length of stay and that it may reflect limited access and availability to rehabilitation services where they live [36]. However, when controlled for other factors in the present study, such as injury severity, geographical location was not a significant cost driver.

In line with factors associated with the total costs at 12 months post-injury, injury-related factors seemed important regarding rehabilitation costs. Being healthy at time of injury was significantly associated with lower rehabilitation costs, indicating that healthy persons require less rehabilitation services following trauma. The number of injuries was significantly associated with rehabilitation cost, and an increase in number of injuries significantly predicted higher rehabilitation costs. Also, severe head injury and severe upper/lower extremity injury were factors significantly associated with higher rehabilitation costs. A previous study demonstrated that severe trauma, and severe head or spine injury increased the odds of being transferred directly from acute ward to specialized rehabilitation units [37]. Moreover, severe upper/lower extremity injury was a significant cost driver associated with higher costs in this study. Previous studies have shown that orthopedic trauma is associated with impaired physical health-related quality of life [1, 38], but that these patients are given little priority with regard to direct transfer from acute care to rehabilitation units [37]. Persons with severe extremity injuries might seek rehabilitation services in the later stages of recovery, such as physical therapy.

Contrary, severe abdominal and severe thorax injury were significantly associated with lower rehabilitation costs. The results showed that of those having a severe abdominal or thorax injury (AIS ≥ 3), fewer patients had severe injuries in other body regions compared to the respective reference groups (AIS < 3). A possible explanation for these results is that those having severe injury in abdomen or thorax need fewer rehabilitation services compared to those not having a severe injuries in these body regions as a higher number of patients not having a severe injury in abdomen or thorax had severe injuries in other body regions, such as head, spine or extremities. Consequently, persons with severe injuries in abdomen or thorax may need less rehabilitation services during the first year after trauma.

### Strengths and limitations

A strength of this study was the inclusion of trauma patients across all ages. Another strength is that we included the utilization and costs for both in-hospital and post-hospital health and rehabilitation services. Further, the use of registry data minimizes loss to follow-up and the potential recall bias when making calculations based on self-reported data. Because we only included health care services linked to trauma-related diagnosis in the registers, an underestimation of utilization and costs may have occurred in this study. Patients might receive services that are reported to the registers using other diagnosis despite the services may have been provided because of trauma-related consequences. However, an exception was made for specialist services, such as ophthalmologist, specialist in ear, nose, and throat, internal medicine specialist, surgeon, psychologist, neuropsychologist, psychomotor physiotherapist, and neurologist as patients often are referred to these specialists due to consequences of trauma, but the services provided might be recorded under other diagnoses. Furthermore, 19 patients (3%) included in this study died during the first 12-months after injury. They may have lower total costs due to not being able to use the health care system for the entire study period, leading to an underestimation of the total costs per patient. However, the vast majority of the patients (97%) survived the first year after injury and we believe that our cost estimates are representative for the included population. It could also be regarded as a limitation that we did not include costs related to loss of productivity and informal health care services provided by family/friends, which may lead to biased picture of the total costs.

In conclusion, this study utilizes registry data in combination with trial data on costs associated with treatment, rehabilitation and follow-up for persons who have been admitted to trauma departments with moderate or severe traumatic injury. The results showed that most costs were related to trauma care provided at the secondary care level, inpatient rehabilitation services, and home- and community-based services. The latter underlines the importance of considering post-hospital costs to get a more holistic picture of the actual costs associated with trauma. Severely injured patients had a higher health care utilization and average cost compared to patients with moderate injury severity. Injury severity factors were the most prominent cost drivers, and number of injuries, severe head, spine, and extremity injuries were significantly associated with higher costs during the first year following trauma. Being healthy at time of injury was significantly associated with reduced costs. The results give an insight into health care utilization and costs in a heterogenous patient group with complex needs and across key subgroups, which can contribute to the planning and provision of treatment and rehabilitation for this patient group.

## Data Availability

The datasets generated and analysed during the current study are not publicly available due to the sensitivity of the material.
